# Methamphetamine facilitates HIV infection of primary human monocytes through inhibiting cellular viral restriction factors

**DOI:** 10.1186/s13578-021-00703-4

**Published:** 2021-11-10

**Authors:** Yu Liu, Feng-Zhen Meng, Xu Wang, Peng Wang, Jin-Biao Liu, Wen-Hui Hu, Won-Bin Young, Wen-Zhe Ho

**Affiliations:** 1 Department of Pathology and Laboratory Medicine, Temple University Lewis Katz School of Medicine, 3500 N Broad St., Philadelphia, PA 19140 USA; 2 Center for Substance Abuse Research, Temple University Lewis Katz School of Medicine, Philadelphia, PA 19140 USA

**Keywords:** Methamphetamine, Human immunodeficiency virus, Interferon-stimulated genes, Monocytes

## Abstract

**Background:**

Methamphetamine (METH), a potent addictive psychostimulant, is highly prevalent in HIV-infected individuals. Clinically, METH use is implicated in alteration of immune system and increase of HIV spread/replication. Therefore, it is of importance to examine whether METH has direct effect on HIV infection of monocytes, the major target and reservoir cells for the virus.

**Results:**

METH-treated monocytes were more susceptible to HIV infection as evidenced by increased levels of viral proteins (p24 and Pr55Gag) and expression of viral GAG gene. In addition, using HIV Bal with luciferase reporter gene (HIV Bal-eLuc), we showed that METH-treated cells expressed higher luciferase activities than untreated monocytes. Mechanistically, METH inhibited the expression of IFN-λ1, IRF7, STAT1, and the antiviral IFN-stimulated genes (ISGs: OAS2, GBP5, ISG56, Viperin and ISG15). In addition, METH down-regulated the expression of the HIV restriction microRNAs (miR-28, miR-29a, miR-125b, miR-146a, miR-155, miR-223, and miR-382).

**Conclusions:**

METH compromises the intracellular anti-HIV immunity and facilitates HIV replication in primary human monocytes.

**Supplementary Information:**

The online version contains supplementary material available at 10.1186/s13578-021-00703-4.

## Introduction

METH is one of the most widely abused illicit drugs among HIV-infected individuals. METH use and HIV infection frequently coexist due to the association of METH use with engagement of high-risk behaviors [1–3]. There is a high prevalence of HIV infection in METH using population [4, 5]. Among men who have sex with men, those who use METH are more susceptible to HIV infection than non-users [6–10]. Clinically, METH use has been implicated in HIV disease progression [11]. Active METH users with HIV infection display higher levels of viral load than non-users [12]. In addition, METH users have delayed viral suppression after initiation of antiretroviral therapy (ART), higher levels of blood HIV RNA, increased frequency of drug resistance mutations and accelerated progression to AIDS [13–17]. METH abuse contributes to CD4^+^ T cells depletion, inflammation/immune activation, and the promotion of HIV entry and disease progression [18].

Cells of monocyte/macrophage lineage are crucial in initial HIV infection and implicated in the immunopathogenesis of HIV disease. Monocytes are among the first and major cell types infected by HIV and serve as reservoirs for the virus. However, unlike tissue macrophages and in vitro monocyte-derived macrophages which are highly susceptible to HIV infection, peripheral blood monocytes are refractory to HIV infection in vivo and in vitro, and only a small percentage of monocytes harbor the virus [19, 20]. Despite of their relative resistance to HIV infection, monocytes are involved in HIV infection of the central nervous system (CNS) as they can bring the virus to the brain [21]. A study reported that HIV-infected monocytes are more likely to cross the blood brain barrier (BBB) as compared to uninfected monocytes [22]. Among HIV-infected METH users, HIV-associated neurocognitive disorders (HAND) are more frequent and severe [23, 24]. A recent study demonstrated that METH could enhance HIV infection of neural progenitor cells, a possible mechanism for the impairment or disruption of neurocognitive functioning in HIV-infected individuals with NeuroAIDS [25]. Several studies showed that elevated extracellular CNS dopamine by METH abuse could facilitate uninfected and HIV-infected CD14^+^CD16^+^ monocytes transmigration across the BBB, resulting in the propagation of viral reservoirs and inflammation in the CNS which contribute to the development of HAND [26, 27]. Thus, it is of great interest to determine the direct impact of METH on susceptibility of peripheral blood monocytes to HIV infection. In addition, it is critical to understand the pathological effects of METH on the specific intracellular innate immunity against HIV in monocytes.

## Results

### METH enhances HIV infection

**Fig. 1:**
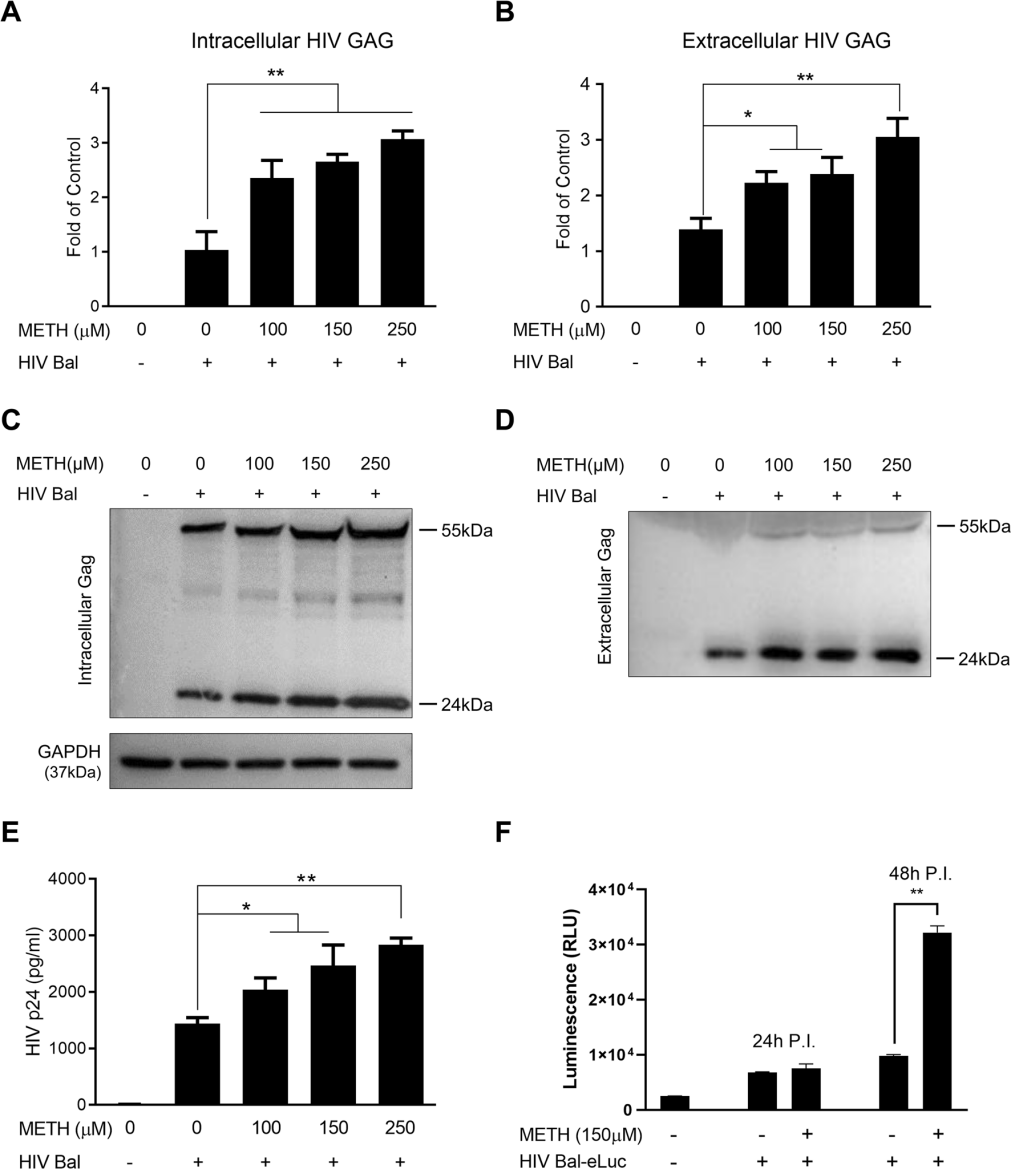
METH enhances HIV infection of primary human monocytes. **A**–**E** Monocytes isolated from human peripheral blood were treated with METH for 24 h and then infected with HIV Bal strain overnight. Cells were washed with PBS three times and cultured in the presence of METH for 72 h. RNAs extracted from cells (**A**) and the cell-free supernatants (**B**) were subjected to the real-time PCR with HIV GAG gene primers. **C**, **D** Proteins of cells and culture supernatants were analyzed by Western blot using the antibodies against HIV proteins (p24 and Pr55Gag) and GAPDH. **E** The cell-free supernatants were subjected to ELISA assay to quantitatively determine p24 protein level. **F** Monocytes were treated with METH (150 μM) for 24 h and then infected with HIV Bal-eLuc overnight. Cells were washed with PBS three times and cultured in the presence of METH for 24 h or 48 h prior to luminescence assay. Data shown were the mean ± SD of three independent experiments with monocytes from three different donors (*P < 0.05, **P < 0.01)

We first examined cytotoxicity effect of METH on monocytes. As shown in Additional file 1: Fig. S1, METH at the concentration as high as 1000 μM had little effect on cell viability. We then studied whether METH could enhance susceptibility of monocytes to HIV infection. As demonstrated in Fig. 1A and B, METH treatment of monocytes dose-dependently increased the expression of both intracellular and extracellular HIV GAG gene expression. In addition, METH-treated monocytes showed higher levels of HIV (p24 and Pr55Gag) proteins than untreated cells (Fig. 1C and D). The enhancing effect of METH on HIV p24 protein production was dose-dependent (Fig. 1E). As shown in Fig. 1F, METH treatment enhanced luciferase activity in HIV Bal-eLuc-infected cells.

### METH suppresses the JAK/STAT signaling pathway

**Fig. 2:**
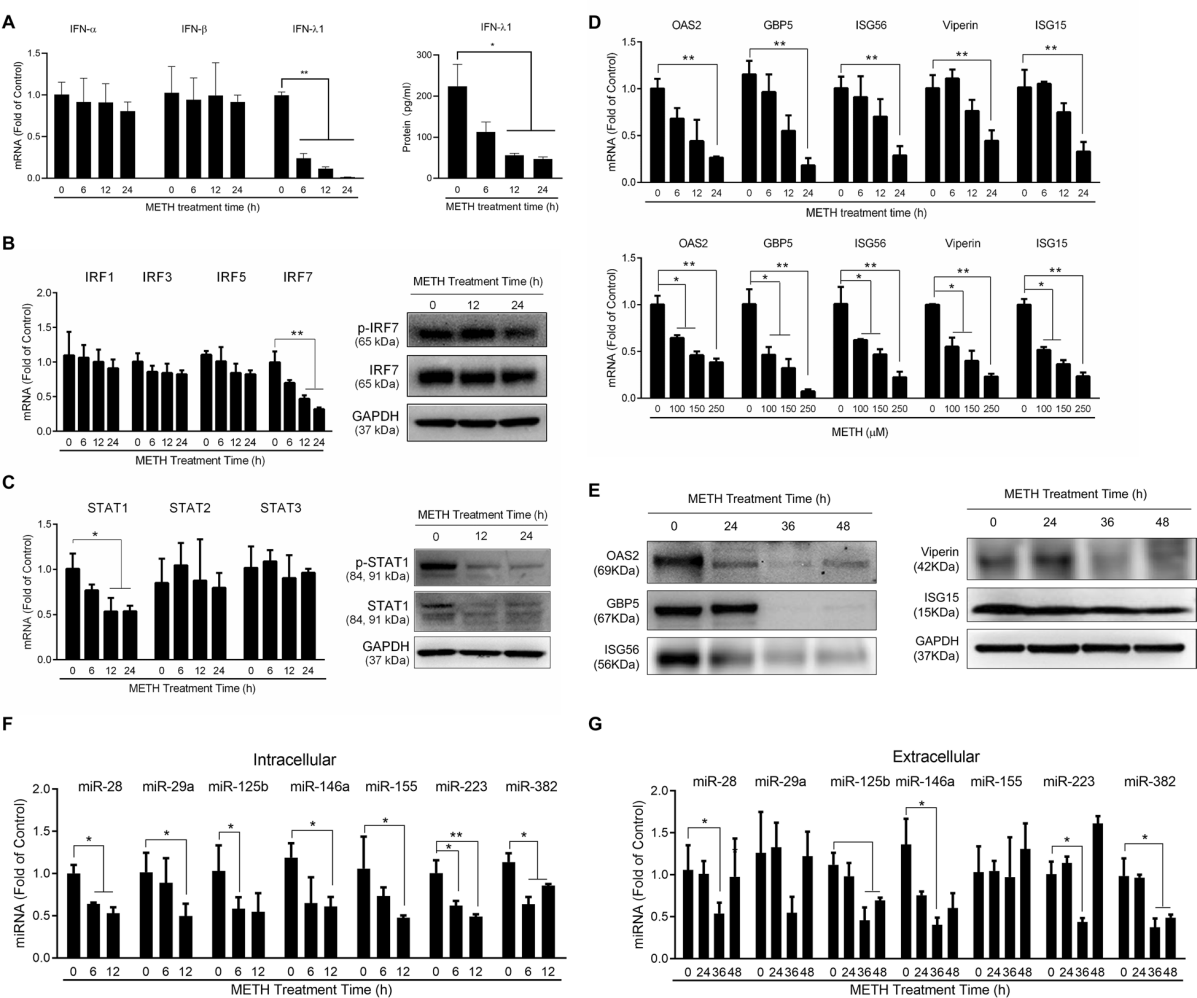
METH inhibits viral restriction factors. **A**–**E** Monocytes from human peripheral blood were treated with METH (150 μM) for the indicated times or at the indicated concentrations for 24 h. **A** The cellular RNAs were extracted and subjected to the real-time PCR for IFN-α, IFN-β, and IFN-λ1 mRNA expression, the culture supernatants were collected and subjected to ELISA for IFN-λ1 protein expression. **B**–**E** The cellular RNAs or proteins were extracted and subjected to the real-time PCR or Western blot assays. **F**, **G** Monocytes were treated with METH (150 μM) for the indicated times. Cellular miRNAs were quantified by the real-time PCR. RNU48 was used as a control gene. miRNAs in the culture supernatants were quantified by the real-time PCR. Synthetic *Caenorhabditis elegans* miRNA-39 (cel-miR-39) was used as a spiked-in control miRNA for normalization. Data shown were the mean ± SD of three independent experiments with monocytes from three different donors (*P < 0.05, **P < 0.01)

To study the mechanisms by which METH enhances HIV infection of monocytes (Fig. 1), we examined the effect of METH on IFNs. As shown in Fig. 2A, while it had little effect on IFN-α/β expression, METH treatment significantly suppressed IFN-λ1 expression in monocytes. In addition, METH inhibited the expression of phosphorylated IRF7 in a time-dependent fashion (Fig. 2B).

IRF7 is a key regulator for both type I and type III IFNs during viral infections [28, 29]. The phosphorylation of IRF7 would directly trigger the transcription of IFNs and the downstream antiviral signaling, including the activation of JAK/STAT pathway and the production of ISGs. We also examined whether METH has a negative impact on the expression of STAT family members including STAT1, STAT2 and STAT3, the crucial factors in JAK/STAT signaling pathway [30, 31]. As shown in Fig. 2C, while METH treatment of monocytes had little effect on STAT2 and STAT3 expression, it significantly inhibited STAT1 expression at both mRNA and protein levels, and reduced the phosphorylation of STAT1. We next examined the effect of METH on the expression of the intracellular antiviral ISGs. As shown in Fig. 2D, METH dose-dependently inhibited the expression of the antiviral ISGs (OAS2, GBP5, ISG56, Viperin, ISG15) at 24 h post-treatment. In addition, the Western blot analysis demonstrated that METH-treated monocytes had lower protein levels of the antiviral ISGs than the untreated cells (Fig. 2E).

### METH inhibits HIV restriction miRNAs

Our earlier study showed that monocytes contain significantly higher levels of the HIV restriction miRNAs than monocyte-derived macrophages, which explains why monocytes are refractory to HIV infection [32]. We thus investigated whether METH negatively influences the expression of the HIV restriction miRNAs in monocytes. As shown in Fig. 2F, METH treatment of monocytes suppressed the expression of the intracellular HIV restriction miRNAs (miR-28, miR-29a, miR-125b, miR-146a, miR-155, miR-223, and miR-382). In addition, we observed lower levels of these HIV restriction miRNAs in the supernatants of monocyte cultures 36 h after METH treatment compared to those in untreated cells (Fig. 2G).

## Discussion

Although METH use has been linked to HIV transmission and infection, its pathological effects on the host cell-mediated specific innate immunity against HIV infection remain to be determined. The earlier studies reported that METH could enhance HIV infection of several cell types, including dendritic cells [33], macrophages [34, 35], CD4^+^ T cells [36, 37], microglia [38], and neural progenitor cells [25]. However, it is unclear whether METH facilitates HIV infection of primary human monocytes. In the present study, we demonstrated that METH treatment of the monocytes significantly enhanced HIV infection/replication at both intracellular and extracellular levels (Fig. 1). To investigate the underlying mechanisms of METH-mediated HIV enhancement in monocytes, we examined that the impact of METH on the expression of IFNs-JAK/STAT signaling pathways. We found that although METH treatment of monocytes had little effect on IFN-α and IFN-β expression, it significantly suppressed IFN-λ1 expression (Fig. 2A). IFN-λ can induce type I IFN-like antiviral response and inhibition of HIV [39, 40]. It is likely that IFN-λ inhibition by METH can result in reduction of ISGs. The following results indicated that METH treatment of the cells down-regulated the expression of the antiviral ISGs, including OAS2, GBP5, ISG56, Viperin, and ISG15 (Fig. 2D and E). These ISGs are known to have the ability to restrict HIV replication at different steps of viral replication cycle [41–45]. For instance, Krapp et al. demonstrated that the expression of GBP5 could interfere with the processing and virion incorporation of the HIV envelope glycoprotein, which remarkably reduce virion incorporation of mature gp120 and enhance virion-associated immature gp160 precursor, leading to the inhibition of HIV infectivity [42]. Okumura et al. showed that ISG15 impaired the interaction between HIV GAG and tumor susceptibility gene 101 (Tsg101), and suppressed HIV virion release [46].

Given that the IFN regulatory factors (IRFs) are responsible for IFN-JAK/STAT signaling pathway and the production of the antiviral ISGs, we also examined the impact of METH on IRFs, particularly IRF1, IRF3, IRF5 and IRF7, the key players in regulating the expression of antiviral ISGs and producing an antiviral state [47]. We found that while METH had little effect on IRF1, IRF3, IRF5, it specifically suppressed IRF7 expression in monocytes at both mRNA and protein levels (Fig. 2B). IRF7 has a key role for the production of both type I and type III IFNs during viral infections [28, 29]. The phosphorylation of IRF7 would directly trigger the transcription of IFNs and the downstream antiviral signaling, including the activation of JAK/STAT pathway. Therefore, it is likely that IRF7 suppression by METH is a possible mechanism for STAT1 inhibition in METH-treated monocytes (Fig. 2C). STAT1 is a crucial regulatory factor in IFNs-mediated induction of antiviral ISGs [48, 49]. While exact mechanism by which METH inhibits IRF and STAT remain to be determined, it is possible that down-regulation of IFN-λ1 has a negative impact on both IRF7 and STAT1 expression.

In addition to the HIV restriction factors of protein nature, a cluster of HIV restriction miRNAs have also been shown to be a contributor to HIV latency in resting CD4^+^ T cells [50, 51]. These cellular miRNAs interact with the 3’-termini of HIV RNA, resulting in the transcriptional inefficiency and post-transcriptional suppression [52, 53]. Our earlier study showed that primary human monocytes expressed much higher levels of miRNAs (miRNA-382, -223, -150, and -28) than monocyte-derived macrophages, and the suppression of these miRNAs facilitates HIV-1 infectivity, which provide direct evidence that HIV restriction miRNAs have a key role in protecting monocytes/macrophages from HIV-1 infection [32]. Several studies reported that METH use altered the miRNA expression in human serum or animal model [54, 55]. We thus determined whether METH could regulate the expression of the miRNAs that are implicated in HIV infection and persistence. Among the cellular miRNAs, the miR-29 family members (miR-29a, miR-29b, and miR-29c) suppress HIV replication by targeting a highly conserved region of HIV [56]. Importantly, our early study documented that the levels of the cellular HIV restriction miRNAs are negatively correlated with susceptibility of monocytes and macrophages to HIV infection [32]. We also reported that ART failed to restore the levels of several HIV restriction miRNAs in PBMCs of HIV-infected men who have sex with men who used METH [57]. Therefore, it is clinically relevant to seek the direct evidence of how METH negatively impacts on the HIV restriction miRNAs. Our observation that METH could significantly suppress the expression of the HIV restriction miRNAs (Fig. 2F and G) provide not only direct evidence for our in vivo finding [57], but also an additional mechanism for METH-mediated enhancement of HIV infection.

It is suggested that METH could facilitate HIV entry into the cells through up-regulation CXCR4 and CCR5, the key coreceptors for HIV entry into target cells [33, 34]. However, in contrast to these earlier studies, we did not observe the enhancing effect of METH on CCR5 expression in monocytes (Additional file 2: Fig. S2). METH also had no significant impact on the expression of CD4. This conflicting finding could be due to the difference in use of different cell types: while we used primary human monocytes, the previous studies used monocyte-derived dendritic cells [33] and macrophages [34] which are more susceptible to HIV infection compared to monocytes [32].

In summary, we demonstrate that METH can enhance HIV infection of primary human in monocytes through the inhibition of the multiple cellular antiviral factors (IFN-λ1, ISGs, and miRNAs). While it is possible that there are additional mechanisms involved in the METH action on HIV enhancement, compromising the intracellular immunity against HIV should be responsible for much of METH-mediated HIV enhancement in monocytes. These findings suggest that METH use is a contributing factor for HIV infection and persistence in monocytes. As HIV-infected individuals are living longer with ART and many of infected individuals are METH users, to further identify the pathological role of METH in HIV-infected reservoir cells is necessary for understanding the mechanisms of HIV persistence and developing strategies for the viral eradication.

## Conclusions

METH compromises the intracellular anti-HIV immunity and facilitates HIV replication in primary human monocytes.

## Materials and methods

### Cells and reagents

Purified human peripheral blood monocytes were obtained from Human Immunology Core at the University of Pennsylvania (Philadelphia, PA, USA). The Core has the Institutional Review Board approval for blood collection from healthy donors. These blood samples were screened for all normal viral bloodborne pathogens and certified to be pathogen free. The protocol of monocyte isolation was described previously [34]. Briefly, after the initial purification, greater than 97% of the cells were monocytes, as determined by nonspecific esterase staining and flow cytometry analysis using monoclonal antibody against CD14, the marker specific for monocytes and macrophages. Freshly isolated monocytes were cultured in 1640 RPMI (Gibco, New York, USA) medium supplemented with 10% fetal bovine serum (Corning, New York, USA), 1% MEM NEAA (Gibco, New York, USA), 1% L-Glutamine (Gibco, New York, USA) and 1% penicillin–streptomycin solution (Lonza, Walkersville, GA, USA). Rabbit antibodies against OAS2, GBP5, ISG56, Viperin, ISG15, IRF7, p-IRF7, STAT1, p-STAT1 and GAPDH were purchased from Cell Signaling Technology (Danvers, MA, USA). Mouse anti-HIV p24 antibody was purchased from Abcam (Abcam, Cambridge, UK). METH was purchased from Sigma Aldrich (St Louis, MO, USA). METH powder was dissolved in sterile endotoxin-free water (HyPure™ Cell culture grade water, GE Healthcare Life Science, Logan, UT, USA) and stored at 4 ℃. Trichloroacetic acid (TCA) and acetone were purchased from Sigma-Aldrich. All antibodies and reagents for flow cytometry assay were purchased from BD Bioscience (BD Bioscience, San Jose, CA, USA).

### HIV infection and METH treatment

HIV Bal strain was obtained from AIDS Reagent Program (NIH, Bethesda, MD), HIV Bal with luciferase reporter gene (HIV Bal-eLuc) was generated by Dr. Won-Bin Young [58]. Freshly isolated and purified monocytes were treated with METH at clinically relevant concentrations [59–62] (0, 100, 150, and 250 μM) for 24 h before being infected with HIV Bal strain (p24 60 ng/10^6^ cells) or HIV Bal-eLuc (p24 60 ng/10^6^ cells) overnight. The cells were then washed three times with plain RPMI to remove any unabsorbed virus and cultured in the presence of METH.

### MTS assay

The cytotoxic effect of METH on monocytes was evaluated by MTS (3-(4, 5-dimethylthiazol-2-yl)-5-(3carboxymethoxyphenyl)-2-(4-sulfophenyl)-2H-tetrazolium, innersalt) assay. Freshly isolated human blood monocytes (2 × 10^4^ cells/well) were placed in 96-well round bottom plates, and treated with different concentrations of METH (0, 100, 150, 250, 400, 600, and 1000 μM) for 96 h. The cells were then incubated with CellTiter 96^®^ AQ_ueous_ One Solution Reagent (Promega Corporation, Madison, WI) containing MTS and phenazine ethosulfate for 4 h at 37 ℃. Absorbance at 490 nm was measured by a plate reader (SpectraMax i3, Molecular Devices, Sunnyvale, CA, USA).

### ELISA

HIV p24 protein levels in monocyte culture supernatants were determined by ELISA kit from Abnova (Taipei, Taiwan) as instructed by the manufacturer. IFN-λ1 protein levels in monocyte culture supernatants were determined by human IL-29 (IFN lambda1) ELISA kit from Invitrogen (Invitrogen, CA, USA) according to the manufacturer’s instruction.

### Flow cytometry assay

Monocytes from human peripheral blood were treated with METH (150 μM) for 24 h. Then the cells were collected and washed with a cell staining buffer prior to staining with PE mouse anti-human CD4 antibody and PE mouse anti-human CCR5 antibody, respectively. PE-isotype IgG antibody-stained cells were used as the negative control. The stained cells were measured by a FACSCanto II (BD Bioscience, CA, USA) and analyzed using FlowJo software (Tree Star Inc., Ashland, OR, USA).

### Western blot assay

Proteins from monocytes and culture supernatants were determined by Western blot assay for viral proteins (p24 and Pr55Gag) expression. Monocytes were lysed with RIPA lysis buffer supplemented with protease/phosphatase inhibitors (Sigma Aldrich, St Louis, MO). Proteins from the culture supernatants were extracted by the TCA/acetone precipitation method. Briefly, 0.5 mL of culture supernatants were precipitated with 0.5 mL of 20% TCA at − 20 °C for 1 h and then centrifuged at 11,500 rpm for 15 min at 4 °C. After three washes with 1 mL of ice-cold acetone, the pellet was lysed with Western blot lysis buffer. The protein concentrations were determined by the bicinchoninic acid (BCA) assay (ChemCruz, Dallas, TX). The blots were incubated with primary antibodies in 5% nonfat milk in PBS overnight at 4 ℃, then washed with PBS containing 0.5% Tween. The blots were further incubated with horseradish peroxidase-conjugated second antibodies at room temperature for an hour, then washed with PBST. The blots were developed with enhanced chemiluminescence (Amersham, Bucks, UK) and then exposed to an iBright 1500 imaging analyzer (Invitrogen, CA, USA).

### RNA and microRNA extraction and quantification

Freshly isolated monocytes in 48-well plates were treated with or without METH (100, 150, and 250 μM) for different time points (0, 6, 12, and 24 h). Total RNAs were extracted with Tri-reagent (Molecular Research Center, OH, USA). RNA (1 μg) was subjected to reverse transcriptase PCR using reagents from Promega (Promega, WI, USA). The cDNA sample was then subjected to the real-time PCR using iQ SYBR Green Supermix (Bio-Rad Laboratories, CA, USA). All values were normalized to GAPDH mRNA. The sequences of oligonucleotide primers used in this study are listed in Table 1. Extracellular miRNAs were extracted from supernatants of monocyte cultures using the miRNeasy Mini Kit (Qiagen, CA, USA). The miRNAs from cells or supernatants were reversely transcribed with miScript Reverse Transcription Kit (Qiagen, CA, USA). The real-time PCR for the miRNAs quantification was carried out with miScript Primer Assays using miScript SYBR Green PCR Kit from Qiagen as previously described [32].

**Table 1:** Primers Pairs

Gene	Orientation	Sequence (5′-3′)
GAPDH	Forward	GGTGGTCTCCTCTGACTTCAACA
	Reverse	GTTGCTGTAGCCAAATTCGTTGT
HIV GAG	Forward	ATAATCCACCTATCCCAGTAGGAGAAA
	Reverse	TTTGGTCCTTGTCTTATGTCCAGAATGC
OAS2	Forward	CAGTCCTGGTGAGTTTGCAGT
	Reverse	ACAGCGAGGGTAAATCCTTGA
GBP5	Forward	CAGGAACAACAGATGCAGGA
	Reverse	TCATCGTTATTAACAGTCCTCTGG
ISG56	Forward	TTCGGAGAAAGGCATTAGA
	Reverse	TCCAGGGCTTCATTCATAT
Viperin	Forward	TGGGTGCTTACACCTGCTG
	Reverse	TGAAGTGATAGTTGACGCTGGT
ISG15	Forward	GGCTGGGAGCTGACGGTGAAG
	Reverse	GCTCCGCCCGCCAGGCTCTGT
IRF1	Forward	TGAAGCTACAACAGATGAGG
	Reverse	AGTAGGTACCCCTTCCCATC
IRF3	Forward	ACCAGCCGTGGACCAAGAG
	Reverse	TACCAAGGCCCTGAGGCAC
IRF5	Forward	AAGCCGATCCGGCCAA
	Reverse	GGAAGTCCCGGCTCTTGTTAA
IRF7	Forward	TGGTCCTGGTGAAGCTGGAA
	Reverse	GATGTCGTCATAGAGGCTGTTGG
STAT1	Forward	CCGTGGCACTGCATACAATC
	Reverse	ACCATGCCGAATTCCCAAAG
STAT2	Forward	CCCCATCGACCCCTCATC
	Reverse	GAGTCTCACCAGCAGCCTTGT
STAT3	Forward	CTGCCCCATACCTGAAGACC
	Reverse	TCCTCACATGGGGGAGGTAG
IFN-α	Forward	TTTCTCCTGCCTGAAGAACAG
	Reverse	GCTCATGATTTCTGCTCTGACA
IFN-β	Forward	GCCGCATTGACCATCTATGAGA
	Reverse	GAGATCTTCAGTTTCGGAGGTAAC
IFN-λ1	Forward	CTTCCAAGCCCACCCCAACT
	Reverse	GGCCTCCAGGACCTTCAGC
CD4	Forward	AGTCCCTTTTAGGCACTTGC
	Reverse	GATCATTCAGCTTGGATGG
CCR5	Forward	CAAGTGTCAAGTCCAATCTA
	Reverse	ACCAAAGATGAACACCAGTG

### HIV GAG gene quantification

HIV GAG gene copy numbers in monocytes or monocytes culture supernatants were determined by the real-time PCR. RNAs from cells or the cell-free supernatants were extracted with Tri-reagent (for tissues, cells cultured in monolayer, or cell pellets) or Tri-reagent (for whole blood, serum/plasma or cell culture supernatant) according to the manufacturer’s instructions, respectively. HIV GAG standards with known copy numbers were used to quantify viral GAG gene expression in the culture supernatants.

### Statistical analysis

Data were expressed as mean ± standard deviation (mean ± SD) of three experiments using monocytes from three different donors. Statistical significance was measured by Student’s t-test using GraphPad Prism Statistical Software (GraphPad Software, La Jolla, USA). *P < 0.05 and **P < 0.01 indicate statistic difference between compared groups.

## Supplementary Information

**Additional file 1: Fig. S1.** Effect of METH on the cell viability of human monocytes. Freshly isolated human monocytes were treated with METH at the indicated concentrations for 96 hours. The cell viability was assessed by MTS assay. Data are showed as the absorbance (490 nm) relative to untreated control, which is defined as 1.0. The results shown were obtained as mean ± SD from three independent experiments with triplicate wells.

**Additional file 2: Fig. S2.** Effect of METH on CD4 and CCR5. (A, B) Freshly isolated human monocytes were treated with METH (150 μM) at the indicated time points. The cellular RNA was subjected to the real-time PCR for CD4 and CCR5 expression. (C, D) Freshly isolated monocytes were treated with METH (150 μM) for 24 h and then collected for the flow cytometry analysis of CD4 and CCR5 protein expression. Data are shown in A and B as mean ± SD from three independent experiments with triplicate wells. Flow cytometry data shown in C and D are the representative pictures of three independent experiments.

## Data Availability

The datasets used and/or analyzed during the current study are available from the corresponding author on reasonable request.
