# Supplementary material for: Methamphetamine facilitates HIV infection of primary human monocytes through inhibiting cellular viral restriction factors
Source: Cell Biosci. 2021 Nov 10;11:194. doi: 10.1186/s13578-021-00703-4 (PMC8579418; doi:10.1186/s13578-021-00703-4)
Supplement: Supplementary file 1 — Additional file 1: Fig. S1. Effect of METH on the cell viability of human monocytes. Freshly isolated human monocytes were treated with METH at the indicated concentrations for 96 hours. The cell viability was assessed by MTS assay. Data are showed as the absorbance (490 nm) relative to untreated control, which is defined as 1.0. The results shown were obtained as mean ± SD from three independent experiments with triplicate wells. [file 13578_2021_703_MOESM1_ESM.docx]

**Supplementary Figure 1**


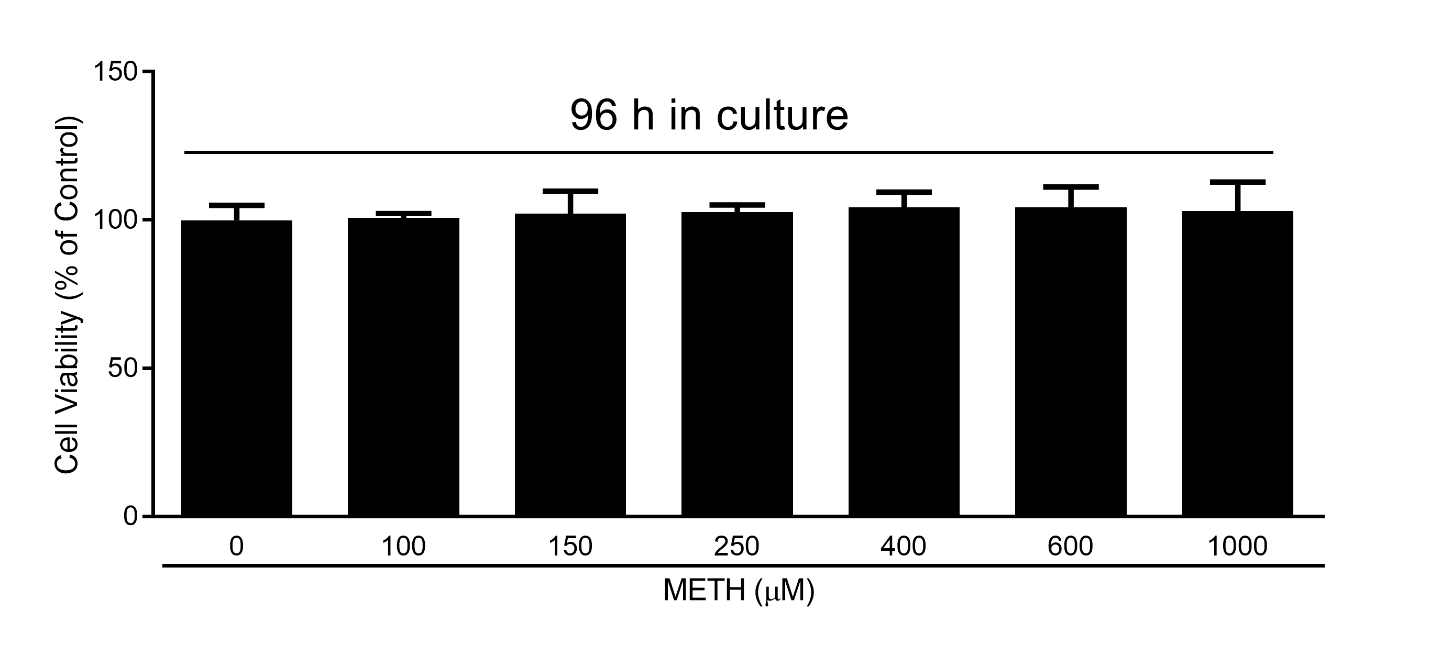


**Supplementary Fig. 1 Effect of METH on the cell viability of human monocytes.** Freshly isolated human monocytes were treated with METH at the indicated concentrations for 96 hours. The cell viability was assessed by MTS assay. Data are showed as the absorbance (490 nm) relative to untreated control, which is defined as 1.0. The results shown were obtained as mean ± SD from three independent experiments with triplicate wells.
