# Supplementary material for: Methamphetamine facilitates HIV infection of primary human monocytes through inhibiting cellular viral restriction factors
Source: Cell Biosci. 2021 Nov 10;11:194. doi: 10.1186/s13578-021-00703-4 (PMC8579418; doi:10.1186/s13578-021-00703-4)
Supplement: Supplementary file 2 — Additional file 2: Fig. S2. Effect of METH on CD4 and CCR5. (A, B) Freshly isolated human monocytes were treated with METH (150 μM) at the indicated time points. The cellular RNA was subjected to the real-time PCR for CD4 and CCR5 expression. (C, D) Freshly isolated monocytes were treated with METH (150 μM) for 24 h and then collected for the flow cytometry analysis of CD4 and CCR5 protein expression. Data are shown in A and B as mean ± SD from three independent experiments with triplicate wells. Flow cytometry data shown in C and D are the representative pictures of three independent experiments. [file 13578_2021_703_MOESM2_ESM.docx]

**Supplementary Figure 2**


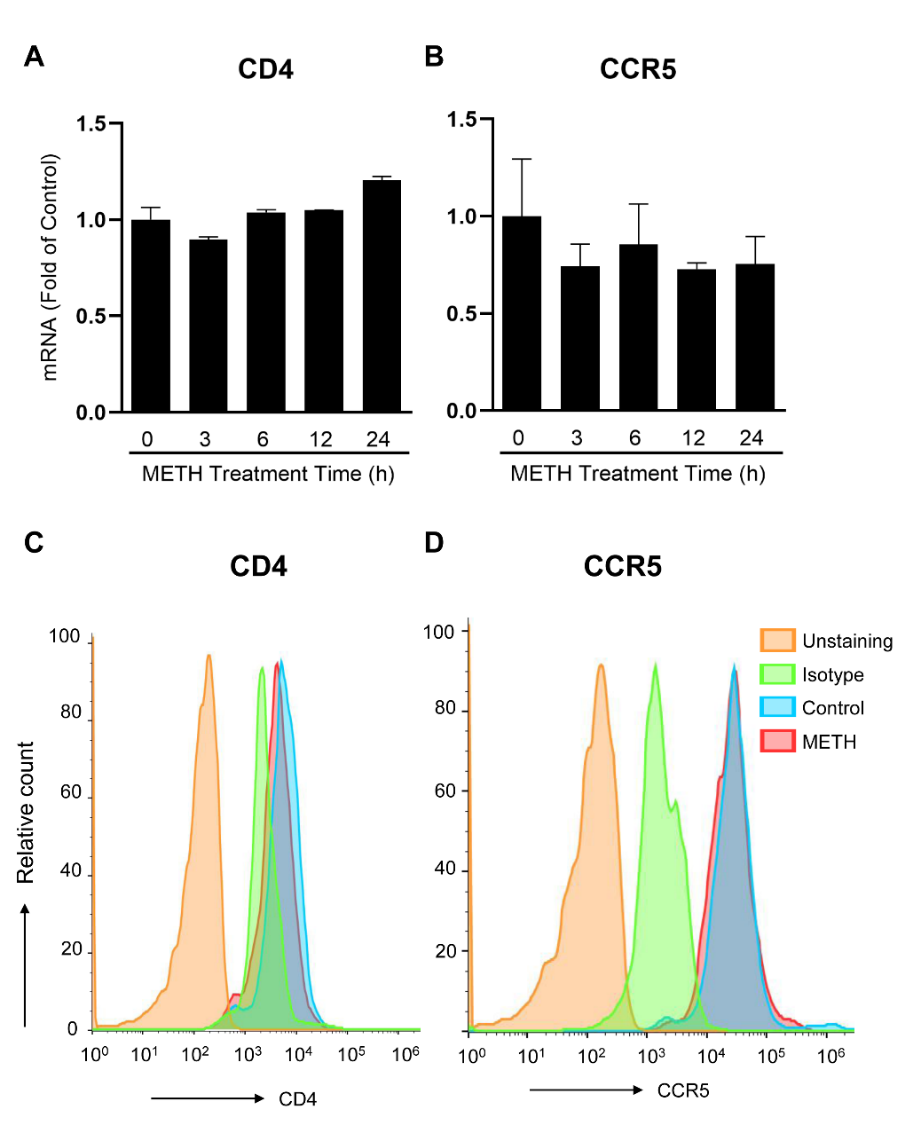


**Supplementary Fig. 2 Effect of METH on CD4 and CCR5. (A-B)** Freshly isolated human monocytes were treated with METH (150 μM) at the indicated time points. The cellular RNA was subjected to the RT-qPCR for CD4 and CCR5 expression. **(C-D)** Freshly isolated monocytes were treated with METH (150 μM) for 24 h and then collected for the flow cytometry analysis of CD4 and CCR5 protein expression. Data are shown in A and B as mean ± SD from three independent experiments with triplicate wells. Flow cytometry data shown in C and D are the representative pictures of three independent experiments.
